# Equivalent Behavioral Facilitation to Tactile Cues in Children with Autism Spectrum Disorder

**DOI:** 10.3390/brainsci11050625

**Published:** 2021-05-13

**Authors:** Girija Kadlaskar, Sophia Bergmann, Rebecca McNally Keehn, Amanda Seidl, Brandon Keehn

**Affiliations:** 1 Department of Speech, Language, and Hearing Sciences, Purdue University, West Lafayette, IN 47907, USA; bergman5@purdue.edu (S.B.); aseidl@purdue.edu (A.S.); bkeehn@purdue.edu (B.K.); 2 Department of Pediatrics, Indiana University School of Medicine, Indianapolis, IN 46202, USA; mcnallyr@iu.edu; 3 Department of Psychological Sciences, Purdue University, West Lafayette, IN 47907, USA

**Keywords:** autism, alerting, tactile processing, auditory processing, attention

## Abstract

The alerting network, a subcomponent of attention, enables humans to respond to novel information. Children with ASD have shown equivalent alerting in response to visual and/or auditory stimuli compared to typically developing (TD) children. However, it is unclear whether children with ASD and TD show equivalent alerting to tactile stimuli. We examined (1) whether tactile cues affect accuracy and reaction times in children with ASD and TD, (2) whether the duration between touch-cues and auditory targets impacts performance, and (3) whether behavioral responses in the tactile cueing task are associated with ASD symptomatology. Six- to 12-year-olds with ASD and TD participated in a tactile-cueing task and were instructed to respond with a button press to a target sound /a/. Tactile cues were presented at 200, 400, and 800 ms (25% each) prior to the auditory target. The remaining trials (25%) were presented without tactile cues. Findings suggested that both groups showed equivalent alerting responses to tactile cues. Additionally, all children were faster to respond to auditory targets at longer cue–target intervals. Finally, there was an association between rate of facilitation and RRB scores in all children, suggesting that patterns of responding to transient phasic cues may be related to ASD symptomatology.

## 1. Introduction

Adaptive allocation of attention to information in one’s environment is critical to the development of cognitive and socio-communicative skills [1,2,3]. For example, shifting attention to surrounding auditory, visual, and tactile inputs enables individuals to recognize different sources of information, extract meaningful information from their environment, and engage in joint attention; thus, it is a necessary skill during social interaction [4]. Nonetheless, individuals with autism spectrum disorder (ASD) typically show early and pervasive impairments in attention [5,6,7]. Furthermore, these differences in attention are often associated with ASD symptomatology [5,8], and have been argued to play a key role in the emergence of the ASD phenotype [9]. 

The alerting network, a subcomponent of attention, is associated with achieving and maintaining a state of high sensitivity to incoming information, and helps humans to recognize and respond to new information [10,11]. Attentional functions subserved by this network have been divided into tonic and phasic components [12,13]. Tonic alertness has been defined as a state of general wakefulness or intrinsic arousal, whereas phasic alertness has been described as a more transient alert state which may be mediated by external stimuli or experimental cues. In typical development, presentation of transient auditory cues either slightly before or simultaneously with visual targets has been shown to impact perception, resulting in faster behavioral responses [14,15,16]. These findings regarding differences in reaction times between cue and no-cue conditions reflect both phasic and tonic alerting [13]. Specifically, transient sensory inputs stimulate alerting responses, thereby improving the processing speed of incoming sensory information after salient events. 

Equivalent phasic alerting has been observed in individuals with ASD compared to their TD peers. For example, in an implicit learning task, Kleberg et al. [17] reported that, similar to TD children, children with ASD showed behavioral facilitation, measured by reduced saccadic reaction times, in a visual disengagement task when transient auditory cues were presented before the visual targets. This is consistent with other prior reports of equivalent alerting in ASD and TD using auditory [18] and visual cues [19,20]. For instance, using the Attention Network Test (ANT) [21], Keehn et al. [19] showed that both children with ASD and TD exhibited similar alerting scores measured by reduced reaction times when target visual stimuli were preceded by visual cues compared to a no-cue condition, indicating similar phasic alerting between the two groups. Further, in Keehn et al. [19], an association was observed between alerting score and the Social Domain score on the Autism Diagnostic Observation Schedule [22], with reduced efficiency of the alerting network being associated with greater social impairments in children with ASD [19]. Together, these findings suggest that children with ASD are able to use auditory and visual cues to facilitate attentional responses within (i.e., visual cues preceding visual targets) [19,20] and across (i.e., auditory cues preceding visual targets) [17,18] modalities, and that differences in alerting network efficiency may be related to the socio-communicative impairments observed in ASD.

Other research investigating the alerting network in ASD has examined whether the response preparation time affects the alerting responses to preceding cues. For example, using an implicit learning task with visual cues and targets, Landry et al. [20] examined children’s behavioral performance in two cue conditions—a variable cue exposure condition (i.e., cues varied in length, with cues of 100, 300, 600, or 1000 ms) and a constant cue exposure condition (i.e., cues were always 100 ms). Additionally, by manipulating the response preparation time (100, 300, 600, 1000 ms) in the constant cue exposure conditions, Landry et al. [20] showed that, while children with ASD exhibited a facilitation effect in response to all stimulus onset asynchronies (SOAs), TD children only showed facilitation at shorter SOAs. These results indicate that variable SOAs might differentially impact behavioral performance in the two groups, warranting further exploration of SOAs for understanding the time course of the alerting response in both ASD and TD children. 

Together, studies utilizing transient visual and auditory cues have shown no differences in alerting in children with ASD compared to TD children. However, questions still remain about the utility of tactile cues in facilitating behavioral responses in children with and without ASD. The present study addresses this gap in the literature by examining the role of tactile cues in facilitating behavioral responses in a cross-modal alerting task in children with ASD compared to their TD peers. We designed a cross-modal task to increase the ecological validity of our study as the majority of information humans receive in everyday life comes from multiple sensory modalities, which may be presented simultaneously (e.g., touching an apple while hearing the word “apple” and seeing an apple) or in close temporal intervals (e.g., receiving a tap on the shoulder slightly before hearing one’s name). In such multimodal situations, especially when related cross-modal stimuli are presented in close temporal intervals, if one fails to attend to any of the stimuli/modalities, or if there are impairments in learning the relationship between two or more stimuli coming from different modalities (e.g., shoulder taps preceding one’s name may indicate upcoming verbal or nonverbal communication), the interlocutor may miss out on an opportunity to receive new information or show slower attentional shifts in response to informative stimuli. This may reduce opportunities to learn novel information and engage in social communication (e.g., failure to anticipate upcoming social interaction following a shoulder tap may result in reduced opportunities to learn and practice social skills such as turn taking, using gestures, maintaining eye contact, etc.). Thus, the current study employed a cross-modal tactile cueing paradigm where touch cues were presented prior to auditory targets.

However, prior studies have shown that individuals with ASD often show hypo- and/or hyper-reactivity in response to tactile stimulation (for a review, see [23]). These differences may result in atypical processing of tactile cues in ASD. For example, hypo-reactivity to touch might result in failure to perceive transient tactile cues generating reduced touch-related behavioral facilitation in ASD. Alternatively, hyper-reactivity may result in increased sensitivity to tactile stimulation. According to the predictive coding theories, which have been used to explain sensory hypersensitivity, individuals with ASD may often fail to contextualize sensory information in relation to prior expectations about the world and such difficulties may be more prominent in uncertain situations [24,25]. Therefore, children with ASD may perform similarly (or better) compared to their TD peers when tactile cues are present, but may show poorer performance in the absence of tactile cues because this violates the (incorrect) assumption that all targets are preceded by cues.

To explore the utility of tactile cues in an alerting task, the present study employed a tactile-cueing paradigm to investigate (1) how tactile cues presented before auditory targets affect accuracy and reaction times in children with ASD and TD, (2) whether increasing the duration between the presentation of tactile cues and auditory targets impacts accuracy and reaction times in children with ASD and TD, and (3) whether behavioral responses in the tactile-cueing task are associated with ASD symptomatology, including sensory responsivity to touch.

For accuracy, we hypothesized that variable cue-target intervals may affect performance especially at longer cue-target intervals where participants will be required to wait for a longer period of time to submit their responses. Specifically, in line with previous research [18], we predicted that the presentation of tactile cues before the target will be associated with an increase in anticipatory responses in all children. Next, for reaction times (RT), we predicted four possible outcomes: (1) if equivalent alerting response to auditory and visual cues is extended to the tactile modality in children with ASD and TD, then children in both groups should show faster reaction times as a result of tactile cues irrespective of the duration between cues and targets; (2) if children with ASD are hypo-reactive to tactile information, then they should show reduced touch-related behavioral facilitation compared to TD children as measured by decreased changes in reaction times in response to tactile cues across all duration-gaps between cues and targets; (3) if children with ASD are hyper-reactive to tactile information, based on the predictive coding theories as mentioned above, we predict that children with ASD may perform similarly (or better) compared to their TD peers when tactile cues are present, but may show poorer performance in the absence of tactile cues because this violates the (incorrect) assumption that all targets are preceded by cues, or, (4) if children with ASD are slower to attend to tactile inputs then they would show reduced facilitation at shorter cue-target intervals and equivalent alerting at longer cue-target intervals where there is more time to process tactile information and submit their responses. 

Finally, given that differences in alerting efficiency and sensitivity to novel sensory information may be related to socio-communicative differences [9,19] and restricted and repetitive behaviors [26,27], and that there exists an association between processing touch and the development of social communication [28,29,30], we hypothesized that reduced touch-related facilitation will be related to greater ASD symptomatology in both ASD and TD children.

## 2. Materials and Methods

### 2.1. Participants

Fifteen 6- to 12-year-old children with ASD (12 male) and fifteen (12 male) age-, sex- and nonverbal IQ-matched TD children participated in the study (Table 1). Clinical diagnoses for the ASD group were confirmed using the Autism Diagnostic Observation Schedule, Second Edition (ADOS-2) [31], the Social Communication Questionnaire (SCQ) [32], and expert clinical judgement according to Diagnostic and Statistical Manual of Mental Disorder-5th Edition criteria (DSM-5). Because all children in the ASD group were able to communicate using fluent speech, they were given the recommended ADOS-2 Module 3. Out of 30 participants, 26 (12 ASD, 14 TD) were right-handed as measured by the Edinburgh Handedness Inventory (EHI) [33]. Participants in the TD group reported no family history of ASD and the absence of clinically significant ASD symptomatology was confirmed using parent report (all t-scores were below 51 on the Social Responsiveness Scale-2) [34]. Eight (7 males) out of 15 children in the ASD group had a caregiver-reported secondary diagnosis of ADHD. No children in the ASD group reported the presence of any other ASD-related medical conditions (e.g., fragile-X syndrome, tuberous sclerosis). Finally, two additional participants in the ASD group were excluded from the final sample due to the refusal to participate in the tactile-cueing task. All participants and their caregivers provided written assent and consent prior to participating in the study. The present research was reviewed and approved by Purdue University Institutional Review Board (IRB).

### 2.2. Standardized Measures 

#### 2.2.1. Autism Diagnostic Observation Schedule, Second Edition (ADOS-2) 

The ADOS-2 [31] is a semi-structured, standardized assessment of communication, social interaction, play, and restricted and repetitive behaviors. All children in the ASD group were administered the ADOS-2 Module 3, which is appropriate for children and adolescents with fluent speech. ADOS-2 calibrated severity scores (CSS) were used as a measure of ASD symptom severity, with higher CSS scores reflecting greater severity [37]. 

#### 2.2.2. Social Responsiveness Scale (SRS-2)

The SRS-2 is a caregiver-report questionnaire that provides a quantitative measure of autism-related traits during the past 6 months. The School-Age form was completed by caregivers in both the ASD and TD groups. The SRS-2 Total T-scores as well as Social Communication and Interaction (SCI) and Restricted Interests and Repetitive Behavior (RRB) scores were used as measures of ASD symptom severity, with higher scores reflecting greater severity.

#### 2.2.3. Sensory Profile-2 (SP-2)

The SP-2 [38] is a caregiver-report questionnaire that assesses everyday sensory processing in 3- to 14-year-olds. Touch and Auditory Sensory Profile scores were used as measures of tactile and auditory sensory processing, respectively.

### 2.3. Experimental Stimuli

#### 2.3.1. Auditory Stimuli 

Auditory stimuli consisted of the vowel sound /a/ generated using the Praat software [39]. The /a/ sound displayed a fundamental frequency of 140 Hz (as it fits within the pitch range of a typical male speaker) [40], and the duration of the vowel was set as 200 ms (similar to the duration of stimuli used in [41]). Auditory stimuli were presented at 60 dB using a central speaker located approximately 60 cm from the participant. 

#### 2.3.2. Tactile Stimuli

**Figure 1:**
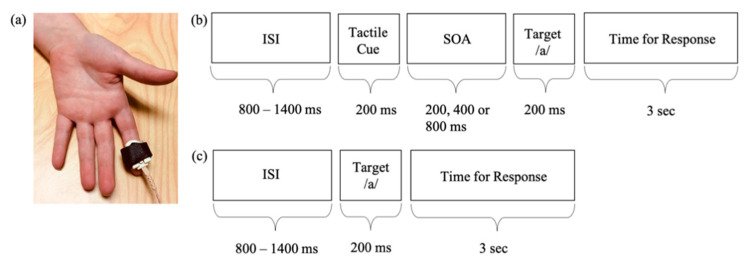
(**a**) Mechanical tactor used to deliver the tactile cues. Illustration of trials with (**b**) and without (**c**) tactile cues. ISI, Interstimulus Interval; SOA, Stimulus Onset Asynchrony.

A custom tactor was used to deliver vibrotactile stimuli to participants’ index fingertip of the non-dominant hand (Figure 1a). Vibrotactile stimuli were delivered on the fingertip to be consistent with the location of the tactile stimuli in the past studies that have examined touch responsivity in individuals with ASD [42,43,44]. Vibrotactile stimuli were presented to the non-dominant hand because participants were instructed to respond with a button press using their dominant hand. Tactile stimuli consisted of vibrotactile stimulation presented at a frequency of 290 Hz. A vibrotactile frequency of 290 Hz was chosen because individuals with ASD have shown differences in tactile responsivity to high-frequency, but not low-frequency, vibrations [42]. Similar to the duration of the auditory stimuli, tactile stimuli also lasted for 200 ms. Finally, each participant’s hand was covered with a towel to mask the sound coming from the tactor.

### 2.4. Procedure

Participants completed a tactile-cuing task in which they were instructed to respond with a button press to the target speech sound /a/. In 75% of the trials, participants received a tactile cue before the target speech sound. Tactile cues were presented at 200, 400, and 800 ms (25% each) prior to the onset of the target speech sound (Figure 1b). The remaining 25% of the trials were presented without the tactile cues (Figure 1c). Participants were informed that on some trials they would feel a “tingle” before the sound, and on some they would not. They were instructed to press the button as quickly as they could only in response to the speech sound. 

Participants first completed a practice round which included a total of 16 trials. During the practice session, all participants received immediate feedback on the computer after each button press informing them about the accuracy of their response (e.g., “correct” if the button was pressed within 3 s after the speech sound; “incorrect” if the button was pressed before the speech sound or if the button was not pressed at all). In case the participants pressed the button before the sound, the computer feedback reminded them to wait for the speech sound. Participants did not receive feedback regarding their reaction times during practice. The experiment included a total of 96 trials divided into 3 blocks of 32 trials each. Participants did not receive any feedback during test trials.

## 3. Results

Independent sample t-tests showed a greater number sensory symptoms in the tactile and auditory domains based on the SP-2 for children with ASD compared to TD children (touch, *t*(28) = 5.15, *p* < 0. 001, d = 1.86; audition, *t*(28) = 7.66, *p* < 0.001, d = 2.63; Table 1). These results confirmed the presence of caregiver-reported aberrant behavioral responses to tactile and auditory stimuli in children with ASD. 

Given our relatively small sample size, non-parametric tests were used (Statistical Package for the Social Sciences (SPSS) Statistics, version 27). Despite the advantages of non-parametric tests with small sample sizes, these tests are considered less powerful than parametric tests. Therefore, we elected to supplement our analysis using Bayesian statistics as Bayes Factor values indicate the strength of evidence in favor of both the null and alternate hypotheses [45,46]. Bayesian analysis was conducted in Jeffreys’s Amazing Statistics Program (JASP) [47] and the null hypothesis was defined as a uniform prior for all tests. 

### 3.1. Accuracy

A Friedman’s test was conducted to examine whether there was a within-subjects main effect of Interval (no cue, 200, 400, 800 ms SOA) on accuracy of responses. Results indicated that there was a within-subjects main effect of Interval on the percentages of accurate responses in all children (χ^2^(3) = 37.15, *p* < 0.001). These results were supported by Bayesian analysis (BF_10_ > 100), indicating strong evidence in support of the finding. Follow-up Wilcoxon signed rank tests showed that accuracy decreased in trials with longer SOAs. All children showed reduced accuracy at 800 ms SOA compared to 400 ms SOA (Z = −3.26, *p* = 0.001), 200 SOA (Z = −3.80, *p* < 0.001), and no cue Intervals (Z = −4.14, *p* < 0.001). Similarly, children showed reduced accuracy at 400 ms SOA compared to 200 ms SOA (Z = −3.32, *p* = 0.001) and no cue Intervals (Z = −3.57, *p* < 0.001). These results were supported by Bayesian analysis (all BF_10_ > 24.01), indicating strong evidence in support of the finding. There were no differences in accuracy between 200 ms SOA and no cue Intervals (Z = −0.65, *p* = 0.51; BF_10_ = 0.19).

Next, Mann–Whitney U tests were conducted to examine between group differences in accuracy at all Intervals. Results showed that the groups did not differ in percentage of accuracy at all four Intervals (no cue: U = 81.50, *p* = 0.15; 200 ms SOA: U = 87, *p* = 0.25; 400 ms SOA: U = 76.50, *p* = 0.12; 800ms SOA: U = 94.50, *p* = 0.45). Bayesian analysis showed inconclusive evidence that the two groups differed in their accuracy at no cue (BF_10_ = 1.02) and 200 ms SOA (BF_10_ = 1.37) Intervals. The two groups did not differ in their accuracy at 400 ms (BF_10_ = 0.91) and 800 ms SOA (BF_10_ = 0.83) Intervals, supporting the results of non-parametric tests. 

### 3.2. Anticipatory Responses 

Given the main effect of Interval on accuracy, a Friedman’s test was conducted to examine whether anticipatory responses—a type of error that occurred when participants pressed the button before the presentation of a target speech sound or within 100ms after the speech sound—were also affected by variable cue target duration (no cue, 200, 400, 800 ms SOA). Results indicated that there was a within-subjects main effect of Interval on the percentages of anticipatory responses in all children (χ^2^(3) = 53.56, *p* < 0.001). These results were supported by Bayesian analysis (BF_10_ > 100), indicating strong evidence in support of the finding. Follow-up Wilcoxon signed rank tests showed that anticipatory responses were most prevalent in trials with longer SOAs. All children showed greater anticipatory responses at 800 ms SOA compared to 400 ms SOA (Z = −3.82, *p* < 0.001), 200 SOA (Z = −4.20, *p* < 0.001), and no cue Intervals (Z = −4.11, *p* < 0.001). Similarly, children showed greater anticipatory responses at 400 ms SOA compared to 200 ms SOA (Z = −3.74, *p* < 0.001) and no cue Intervals (Z = −3.55, *p* < 0.001). These results were supported by Bayesian analysis (all BF_10_ > 87.65), indicating strong evidence in support of the finding. There were no differences in anticipatory responses between 200 ms SOA and no cue Intervals (Z = −0.51, *p* = 0.60; BF_10_ = 0.21).

Next, Mann–Whitney U tests were conducted to examine between group differences in anticipatory responses at all Intervals. Results showed that the groups did not differ in percentage of anticipatory responses at all four Intervals (no cue: U = 96.50, *p* = 0.26; 200 ms SOA: U = 112.50, *p* = 1.0; 400 ms SOA: U = 85.50, *p* = 0.24; 800 ms SOA: U = 110.50, *p* = 0.93). These results were supported by Bayesian analysis (all BF_10_ < 0.68).

### 3.3. Reaction Time

**Figure 2:**
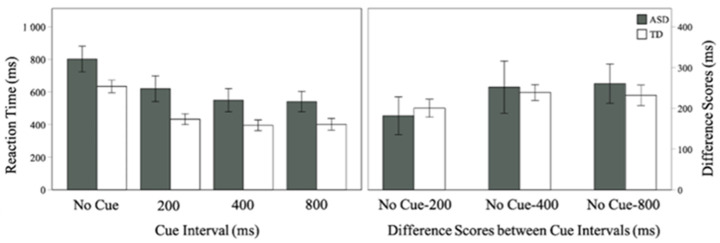
Reaction times showing both groups are faster to respond when tactile cues are present (left) and difference scores between touch cue intervals (right). Error bars represent ±1 SE of the mean.

A Friedman’s test was conducted to examine whether median reaction times were affected by touch cue Intervals (no cue, 200, 400, 800 ms SOA). Results showed that there was a within-subjects main effect of Interval on reaction time in all children (χ^2^(3) = 54.20, *p* < 0.001). These results were supported by Bayesian analysis (BF_10_ > 100), indicating strong evidence in support of the finding. Follow-up Wilcoxon signed rank tests showed that reaction times were reduced (i.e., faster responses) when SOAs were longer. Compared to no cue trials, median RTs were reduced for 200 ms SOA, Z = −4.67, *p* < 0.001, 400 ms SOA, Z = −4.63, *p* < 0.001, and 800 ms SOA, Z = −4.74, *p* < 0.001. Compared to 200 ms SOA, median RTs were reduced for 400 ms SOA Z = −2.97, *p* = 0.003, and 800 ms SOA Z = −3.61, *p* < 0.001. These results were supported by Bayesian analysis (all BF_10_ > 3.14) indicating substantial evidence in support of the finding. There were, however, no differences between median RTs between 400 ms and 800 ms SOA (Z = −0.56, *p* = 0.57; BF_10_ = 0.19; Figure 2). 

Next, Mann–Whitney U tests were conducted to examine between group differences in reaction times at all Intervals. Results showed that the groups did not differ in reaction times at all four Intervals (no cue: U = 76, *p* = 0.13; 200 ms SOA: U = 72, *p* = 0.09; 400 ms SOA: U = 73, *p* = 0.10; 800 ms SOA: U = 71, *p* = 0.08). Bayesian analysis showed inconclusive evidence that the two groups differed at all four Intervals (no cue: BF_10_ = 1.31; 200 ms SOA: BF_10_ = 1.93; 400 ms SOA: BF_10_ = 1.42; 800 ms SOA: BF_10_ = 1.31). 

To further explore whether the two groups differed in their variability in reaction times, we calculated the Coefficient of Variation and Standard Deviation (SD) of RTs for each subject at all cue intervals. A Friedman’s test showed that there was no within-subjects main effect of Interval on Coefficient of Variation for RTs in all children (*X*^2^(3) = 1.96, *p* = 0.58). For SD, however, there was a significant within-subjects main effect of Interval in all children (*X*^2^(3) = 25.48, *p* < 0.001). Follow-up Wilcoxon signed rank tests showed that SD in RTs was greater at no cue Interval compared to 200 ms SOA (Z = −4.47, *p* = 0.01), 400 ms SOA (Z = −2.91, *p* = 0.004), and 800 ms SOA (Z = −3.46, *p* = 0.001). There were no differences in SD among the three touch cue intervals (all *p_s_* > 0.086). Finally, Mann–Whitney U tests showed that the two groups differed in their Coefficient of Variation and SD at four Intervals (i.e., no cue, 200, 400, 800 ms; all *p_s_* < 0.03). Bayesian analysis supported these results. 

### 3.4. Difference Score Analysis for Reaction Times at Variable Cue–target Intervals

Difference scores were calculated by subtracting median RTs in each of the three tactile-cueing Intervals from median RTs in the no-cue condition (i.e., RT_No Cue_—RT_200 SOA_; RT_No Cue_—RT_400 SOA_; RT_No Cue_—RT_800 SOA_). A Friedman’s test showed a main effect of Interval (χ^2^(2) = 16.26, *p* < 0.001). Follow up Wilcoxon tests showed that when presented with touch cues, facilitation was greater at 400 (Z = −2.97, *p* = 0.003) and 800 ms SOA (Z = −3.50, *p* < 0.001) compared to 200 ms SOA. Bayesian analysis supported these findings (BF_10_ = 3.14; BF_10_ > 100, respectively). The facilitation effect did not differ between 400 and 800ms SOA (Z = −0.456, *p* = 0.57; BF_10_ = 0.19). Mann–Whitney U tests and Bayesian independent samples t-tests showed that the two groups did not differ in behavioral facilitation at all SOAs (all *p_s_* > 0.39; all BF_10_ < 0.38).

#### Difference Score x Interval Slope

To examine the rate of behavioral facilitation across SOAs, slopes were extracted from difference scores across all three touch–cue Intervals. Flat slopes reflect less time required to reach full facilitation benefits as a result of tactile cues presented at various intervals, whereas steeper positive slopes would reflect longer time required to reach maximum benefit from tactile cue intervals. Results of the Mann–Whitney U test showed that slopes (U = 70, *p* = 0.08) did not differ significantly between the two groups. Bayesian analysis showed inconclusive evidence that the slopes differed between ASD and TD groups (BF_10_ = 1.22). 

### 3.5. Correlation between Reaction Time and ASD Symptomatology

Spearman’s correlations examined the association between performance in the tactile-cueing task (measured by reaction time difference scores and slopes) and ADOS-2 severity scores along with SRS-2, and tactile and auditory SP-2 scores. In order to reduce the number of correlations run for reaction time, the mean of the three difference scores (explained above) was used as a measure of performance facilitation on the tactile-cueing task. Spearman’s correlations showed no associations between reaction times and ASD symptomatology for both groups (all *p_s_* > 0.10). For the ASD group, there was a significant positive correlation between slope and ADOS-2 Restricted Interests and Repetitive Behavior score (*r*(15) = 0.52, *p* = 0.04). Additionally, there was a positive correlation between slope and SRS-2 Restricted Interests and Repetitive Behavior score for all children (*r*(30) = 0.41, *p* = 0.02).

## 4. Discussion

The goal of this study was to investigate behavioral facilitation associated with tactile cues in children with and without ASD in a tactile-cueing paradigm. Past research has revealed that children with ASD may show equivalent alerting in behavioral tasks in which a target stimulus is preceded by cues that fall within [19,20] or outside [17,18] the target modality. These studies showing similar phasic alerting responses in ASD and TD have mainly used auditory and visual cues to investigate this response within and across modalities. We designed a task that specifically included tactile cues because of the role that touch plays in facilitating attention [48,49] and because individuals with ASD often show differences in processing tactile stimuli [23] that may result in atypicalities in effectively using informative tactile cues to benefit their behavioral responses compared to TD individuals. 

In the current study, we examined (1) whether transient tactile cues, presented before an auditory target, impact behavioral responses measured by accuracy and reaction time in children with and without ASD, and (2) whether changes in the duration (SOAs) between tactile cues and auditory targets impact the accuracy and reaction time of behavioral responses in children with and without ASD. Additionally, because previous research has shown links between the alerting network and ASD symptomatology [19], our third aim was to investigate whether behavioral responses in the tactile-cueing task are associated with ASD symptomatology in children with ASD and TD. 

Our results indicated that not only the presence of tactile cues, but also the duration between tactile cues and auditory targets, impacted accuracy in both the ASD and TD groups. Specifically, all children exhibited higher accuracy in conditions with no touch-cues and at 200 ms SOA. Children were more likely to make errors (i.e., anticipatory responses) when touch-cue intervals were longer. However, the percentage of correct and anticipatory responses did not differ between children with and without ASD at any of the intervals, suggesting similar error patterns in ASD and TD at all touch-cue intervals. 

Our finding of increased error rate in conditions with longer touch-cue intervals is partially consistent with the findings of Raymaekers et al. [18], who showed increased anticipatory errors when salient auditory cues were presented before visual targets in children with and without ASD. However, it should be noted that the current paradigm differed slightly from that of Raymaekers et al. [18]. In the current study, we systematically examined the effect of variable cue-target interval on participants’ responses, whereas Raymaekers et al. [18] did not discuss differences in interstimulus interval in relation to error rate. As observed in Raymaekers et al. [18], if accuracy and anticipatory responses were merely affected as a result of presenting cues before the target, then there would have been a uniform increase in error rate at all three touch-cue intervals. However, this was not the case; anticipatory errors were more likely at 400 and 800 ms SOA, but not at 200 ms SOA. Given the evidence, we extend the findings of Raymaekers et al. [18] to an audio-tactile paradigm, suggesting a modality-independent anticipatory effect that may be dependent on the duration between cues and targets. In sum, anticipatory errors may reflect failure of inhibiting a response as a result of encountering cues that systematically indicate upcoming targets, and may be more prominent when participants are required to wait longer to be presented with a target and submit their responses. Finally, our results suggest that the patterns of response inhibition were similar across the two groups.

Presentation of transient tactile cues before auditory targets resulted in faster reaction times in all children, suggesting similar alerting in ASD and TD. These findings are consistent with previous studies that have shown evidence of faster reaction times when within or across-modality cues are presented shortly before the target stimuli [15,16,17,18,19]. We extend these findings to audio-tactile modality and suggest that efficient phasic alerting may also be observed in multimodal settings in ASD and TD. Our findings also revealed that variable touch-cue intervals affected reaction times in both groups. Specifically, although the facilitation effect was present at 200 ms, it reached its peak at 400 ms, after which it plateaued in both groups. These results add to previous research by Landry et al. [20], in which children with and without ASD showed attenuated reaction times as SOAs between cues and targets increased; longer intervals between cues and targets may have provided children with more time to prepare for a response resulting in faster RTs following the presentation of the target compared to shorter cue-target intervals. 

Additionally, our variability analysis suggested that standard deviations in individual reaction times were greater at no cue intervals for all children compared to when participants were presented with tactile cues prior to the target. Moreover, children in the ASD group showed more variability in their reaction times compared to their typically developing peers. A recent review has suggested that RT variability in individuals with ASD is increased when the sample includes participants with co-morbid ADHD symptoms, and that the variability may reflect differences in arousal levels [50]. It is possible that the parent-reported co-morbid ADHD diagnosis in half of our ASD sample might explain some of the variability observed in children’s individual RTs. However, given our relatively small sample size, a comparison of ASD only vs. ADHD+ASD participants would be beyond the scope of this analysis, but should be a focus for future studies.

Given these results, we argue that, similar to visual and auditory modalities, tactile cues may also impact the alerting network similarly in children with ASD and TD. Additionally, it is possible that the predictable nature of events in touch-cue trials (i.e., presentation of touch cues → waiting period lasting between 200 and 800 ms → presentation of auditory target followed by response) facilitated behavioral performance in all children. Although the present task included variable cue-target intervals as well as 25% of trials with no cues introducing some level of uncertainly regarding the onset of the target, the presence of cues alone always indicated impending targets, making trials with tactile cues more predictable compared to no-cue trials. For instance, every time a participant encountered a tactile cue, it signaled to the participant that a target sound was about to be presented in a brief period of time. This predictable course of events in cued trials may have assisted participants in learning the temporal relationship between cues and targets (i.e., a target will always be presented after a 200 to 800 ms window following the cue). Therefore, along with the efficiency of the alerting network, implicitly learning the temporal pattern between cues and targets may have increased response readiness in all children, resulting in faster reaction times in cued trials compared to no-cue trials. The argument in favor of predictability of events facilitating behavioral responses is consistent with previous findings, suggesting possible links between predictive events and efficient cognitive processing (for a review, see [51]). 

To our knowledge, this is the first study to examine how touch may serve as an informative cue to facilitate behavioral performance in ASD. Differences in tactile processing are a common associated feature in individuals with ASD [23,52], which may affect how touch is perceived and used in various settings. Contrary to parent-reported sensory differences in our ASD group, our results of the tactile-cueing task allow us to argue against an impairment in sensory or attentional processing of tactile stimuli in ASD. In particular, the present findings do not indicate hypo-reactivity to tactile cues in ASD, as a disruption at this level would have resulted in no facilitation effects in any of the three touch-cue intervals. However, this was not the case. Similarly, our results do not suggest an impairment in attending to tactile cues, because if attention to touch were impaired in ASD, then children in this group would have benefitted only from longer cue-target intervals. However, present results showed that facilitation effects were present even at the shortest cue-target intervals in children with ASD. The discrepancy between parent-reported sensory symptoms in the ASD group and children’s performance in the tactile-cueing task may be attributed to differences between responding to non-social experimentally controlled stimuli as opposed to everyday sensory experiences that children encounter in the outside world. We discuss this argument in greater detail in the limitation section below.

Although non-parametric tests indicated that children in both groups showed equivalent reaction times at all cue intervals, Bayesian statistics indicated anecdotal evidence that children with ASD were slower to respond to auditory targets at all four intervals. Consideration of Bayesian results here is important because in frequentist statistics, results with non-significant p-values do not, by default, confirm the null hypothesis, but instead only show inconclusive evidence to reject the null hypothesis [53]. Because Bayesian analysis only showed anecdotal evidence that the two groups differed in their reaction times, it becomes imperative to replicate these findings with larger sample sizes to examine the generalizability of our findings.

Our correlation analysis showed that steeper slopes were associated with greater RRB scores for children in the ASD group as well as for both groups combined. Steeper slopes may reflect longer intervals to reach maximum facilitation benefit of tactile cues presented at variable intervals. We argue that children who fail to respond efficiently to novel transient phasic cues may be more likely to show difficulties in following new sets of rules as a result of some of the core RRB features, such as insistence on sameness and rigidity or inflexibility in applying rules [54,55,56]. While there was a correlation between ASD symptomatology and rate of facilitation, contrary to our hypothesis, there was no association between overall behavioral facilitation to touch cues and measures of ASD symptomatology in ASD and TD children. Our correlational analyses are in contrast with past research that reported links between alerting in response to non-social visual cues and social domain scores of the ADOS [19]. These contradictory results could be related to the nature of the modality. For example, the association between alerting responses to non-social cues and reciprocal social skills could be more prevalent in the visual modality compared to the tactile modality. Because touch is inherently a social signal [29,57] and social touch has been shown to facilitate learning [58], future studies should aim to examine links between alerting in response to social touch and ASD symptomatology, as this correlation may be more robust compared to examining the association between non-social tactile vibrations on the fingertip and ASD symptomatology. 

This study is not without limitations. Our sample was relatively small as a result of limited data collection due to COVID-19. Further, due to task-related demands, our sample included only high-functioning children with ASD and TD. Our sample, therefore, may not be adequately representative of a heterogenous sample of ASD. Additionally, half of our participants in the ASD group also had a parent-reported secondary diagnosis of ADHD, which may have impacted their overall performance on the tactile cueing task. Because prior studies have shown altered alerting in ADHD [59,60,61], future large-scale studies are needed to further examine alerting differences among ASD only, ADHD only, and ASD+ADHD groups.

Next, while the current results showed equivalent alerting in ASD and TD using a behavioral paradigm, prior research has shown atypical phasic alerting and arousal in individuals with ASD using psychophysiological, psychophysical, and electrophysiological measures [9,62,63,64]. For example, an early N1c Event Related Potential (ERP) component that reflects automatic attentional capture has been found to be diminished in amplitude in ASD compared to controls [65,66]. Studies examining skin conductance and heart rate add to these findings by showing atypical levels of arousal in ASD [62,67,68]. Therefore, future studies should employ a multi-method approach to study how differences in perception and/or attention to sensory stimuli as well as internal arousal levels may impact alerting in typical and atypical development.

Although we improved ecological validity by presenting a cross-modal tactile-cueing task, the nature of the tactile cues in our study do not represent the heterogeneity of touches experienced in the outside world. For instance, our tactile stimuli were experimentally controlled non-social vibrations on the fingertip, whereas touches used to facilitate alerting in real-world settings may include a variety of touches (e.g., tap, brush, or tickle), locations (e.g., touch on the shoulder, arm, or leg), and may also show a range of communicative intents and are more dynamic (e.g., touch to get attention or convey affect) [57,69,70]. Individuals presented with touch in non-experimental settings are, therefore, required to respond to a variety of aspects of that touch while learning cross-modal links between touch and other modalities that are presented during that interaction. The crucial difference between the quality of experimentally controlled non-social touch and novel and dynamic touch experienced in the outside world, therefore, warrants further exploration to understand how touch-related alerting in everyday life may contribute to the ASD phenotype. 

Together, results pertaining to accuracy and reaction time indicate that the presence of transient cues may result in faster, but somewhat less accurate, responses in typical and atypical development. These results may indicate challenges in suppressing the arousal effect elicited by transient cues, especially when cue-target intervals are longer. Although a few errors due to difficulty in arousal suppression may be typical, an excessive amount may indicate impulsivity. Our results, therefore, provide a rationale for exploring a possible relationship between errors associated with alerting responses and impulsivity in ASD as well as in other neurodevelopmental disorders such as ADHD. Next, in the present study, participants in the ASD group showed intact alerting responses despite showing higher parent-reported sensory symptoms, suggesting that alerting responses may not be impacted by tactile or auditory sensory reactivity differences. Future studies should evaluate whether sensory reactivity differences impact other subcomponents of attention such as the orienting and the executive functioning networks.

In sum, our results suggest intact alerting in individuals with ASD in the context of tactile cues. However, more research is needed to determine whether these findings may be generalized to tactile situations encountered in the outside world, and whether tactile cues may benefit individuals with ASD in various situations. Finally, given our results of equivalent alerting in ASD and because the study of attention in ASD has consistently documented that differences in attentional disengagement may contribute to the development of the ASD phenotype [9], future studies should examine the role that tactile cues may play in facilitating disengaging and reorienting of attention in addition to studying the efficacy of alerting to various tactile stimuli.

## 5. Conclusions

Our findings suggest that children in both the ASD and TD groups show equivalent phasic alerting in response to transient tactile cues. Additionally, patterns of responding to transient phasic cues may be associated with ASD symptomatology. These results have implications in everyday social interactions, where novel and dynamic touches are naturally used as cues to facilitate alerting before a variety of auditory or visual stimuli are presented. 

## Figures and Tables

**Table 1:** Participant demographics.

	ASD	TD	Statistic	*p*
*N* (M:F)	15 (12:3)	15 (12:3)	χ^2^(1) = 0.00	1.0
Age (years)	10.03 (1.88); 6.17–12.58	9.79 (1.45); 7.55–12.53	*t*(28) = 0.38	0.70
Handedness (R:L)	12:3	14:1	χ^2^(1) = 1.15	0.28
Verbal IQ	96 (22); 67–126	117 (11); 94–135	*t*(28) = −3.40	0.002
Nonverbal IQ	105 (20); 70–136	116 (16); 89–144	*t*(28) = −1.63	0.11
ADOS-2				
Social Affect	11 (5); 4–20	-	-	-
Repetitive Behavior	3 (2); 1–6	-	-	-
Severity Score	8 (2); 4–10	-	-	-
Sensory Profile-2				
Touch Raw Score	23 (9); 5–41	10 (4); 0–15	*t*(28) = 5.15	<0.001
Auditory Raw Score	27 (7); 15–38	12 (4); 2–21	*t*(28) = 7.66	<0.001

*Note.* IQ determined using the Wechsler Abbreviated Scale of Intelligence, Second Edition (WASI-II) [35] or the Differential Ability Scale, Second Edition (DAS-II) [36]. Mean (SD); range.

## Data Availability

All data generated and/or analyzed during this study are available from the corresponding author upon reasonable request.
